# circ-NOLC1 inhibits the development of cervical cancer by regulating miR-330-5p-PALM signaling axis

**DOI:** 10.1186/s41065-025-00478-5

**Published:** 2025-06-18

**Authors:** Yali Duo, Cong Kang, Lei Zheng, Jing Wang, Zhongjie Liu, FengLing Bi, Lei Qiu, Ning Zhao

**Affiliations:** 1 Department of Central Laboratory, Harrison International Peace Hospital, 180 Renmin East Road, Hengshui, Hebei 053000 China; 2 Department of Gynecology, Harrison International Peace Hospital, 180 Renmin East Road, Hengshui, Hebei 053000 China; 3 Department of Pathology, Harrison International Peace Hospital, 180 Renmin East Road, Hengshui, Hebei 053000 China

**Keywords:** Cervical cancer, Circular RNAs, circ-NOLC1, miR-330-5p, PALM

## Abstract

**Background:**

Recent studies have increasingly demonstrated that circular RNAs (circRNAs) play significant roles in the occurrence and progression of cervical cancer (CC). In CC, circRNAs act as ceRNAs by sponging miRNAs to regulate genes associated with proliferation, migration, and apoptosis, exhibiting both promoting and inhibiting effects on tumor progression. The aim of this study was to clarify the role of hsa_circ_0019686 (named circ-NOLC1) in CC.

**Methods:**

By conducting an online GEO2R analysis of the expression profile GSE113696 in the GEO database, circ-NOLC1 was selected. The expression levels of circ-NOLC1 in CC cell lines were measured using real-time quantitative PCR (RT-qPCR). The role of circ-NOLC1 in CC was validated through both in vitro and in vivo gain-of-function assays. Bioinformatic analysis, combined with luciferase reporter and RNA Immunoprecipitation (RIP) assays, confirmed that circ-NOLC1 acts as a sponge for miR-330-5p and regulates the expression of paralemmin-1 (PALM). The role of the circ-NOLC1-miR-330-5p-PALM signaling axis in CC was elucidated through the rescue experiments. Relative gene expression levels were measured using RT-qPCR, while relative protein levels were assessed through immunohistochemistry (IHC). CCK-8, wound healing, Transwell, and flow cytometry assays were employed to evaluate CC cell proliferation, migration, and invasion, respectively.

**Results:**

The expression levels of circ-NOLC1 were dramatically downregulated in CC cells (*P* < 0.001). Up-regulation of circ-NOLC1 significantly inhibited cell proliferation (*P* < 0.001), migration (*P* < 0.01) and invasion (*P* < 0.01), while promoting cell apoptosis (*P* < 0.001). In vivo studies showed that up-regulation of circ-NOLC1 suppressed tumor growth (tumor volume: *P* < 0.001; tumor weight: *P* < 0.01). Additionally, miR-330-5p was found to be up-regulated in CC (*P* < 0.001), whereas PALM was downregulated in CC (*P* < 0.001). The up-regulation of circ-NOLC1 inhibited the expression of miR-330-5p (*P* < 0.001) and enhanced the expression of PALM (*P* < 0.001). Rescue experiments further demonstrated that the up-regulation of circ-NOLC1 inhibited CC cell proliferation (*P* < 0.001), migration (*P* < 0.001), invasion (*P* < 0.001), while promoting apoptosis (*P* < 0.001) through the regulation of the miR-330-5p-PALM pathway.

**Conclusion:**

The circ-NOLC1 inhibits CC development through regulating the miR-330-5p-PALM signaling axis. This finding reveals a novel mechanism and identifies potential therapeutic targets, emphasizing the necessity for further regulatory studies and clinical validation.

## Introduction

Each year ~ 500,000 individuals worldwide are diagnosed with cervical cancer (CC), making it one of the most prevalent gynecologic malignancies [1]. The incidence rate of CC has been steadily increasing, posing a significant threat to women’s lives and health, particularly in underdeveloped countries [2, 3]. Preventing and controlling CC is challenging due to a combination of genetic, environmental, and viral factors [4]. Among these contributing factors, high-risk HPV (HR-HPV) infection plays a central role [5]. Despite improvements in HPV screening and vaccination methods, the morbidity and mortality rates associated with CC continue to rise [6]. Currently, the treatment for CC primarily relies on surgery and radiotherapy; however, these options are severely limited for patients with advanced or recurrent disease [7]. Therefore, there is an urgent need to identify new therapeutic targets for CC.

CircRNA is a novel class of non-coding RNA characterized by covalently closed loops [8]. Based on these molecular properties, circRNAs have been found to possess numerous potential biological functions. For example, circRNAs can act as sponges for microRNAs (miRNAs), inhibiting the ability of miRNAs to regulate mRNAs and thereby participating in the regulation of various physiological and pathological processes [9]. Additionally, circRNAs can interact with RNA-binding proteins (RBPs) to modulate the transcription of their linear parent genes [10]. Most circRNAs are predominantly located in the cytoplasm, where they can enhance the expression levels of downstream genes through competitive adsorption of miRNAs [11]. Recently, the roles of circRNAs in various cancers have been increasingly elucidated, including CC [12]. For example, circRNA_101996 promotes the progression of CC by regulating the miR-1236-3p-TRIM37 signaling pathway [13]. CircEPSTI1 accelerates the pathological processes associated with CC by inhibiting ferroptosis [14]. The downregulation of circRNA_0000285 suppresses CC cell proliferation and facilitates apoptosis [8]. Additionally, circ-CDK6 inhibits the metastasis of CC cells by competitively binding to miR-449a [15]. Collectively, these studies suggest that circRNAs play crucial roles in the development and progression of CC. Analogously, bioinformatics-driven approaches have identified key genes and pathways in other chronic diseases. For instance, Dasgupta et al. employed machine learning to uncover shared genetic signatures between lung cancer and gastroesophageal reflux disease, highlighting the matrix remodeling pathway and hub genes like MMP7 [16]. Cheng et al. used GEO2R and DAVID to screen ulcerative colitis-specific hub genes (GNG11, GNB4) and validated the PI3K/Akt pathway in inflammatory bowel disease [17]. Similarly, Kakar et al. integrated GSE62232 dataset analysis to identify CDK1, CCNB1 as prognosis-related genes in hepatocellular carcinoma [18]. These studies demonstrate the utility of bioinformatics in deciphering disease-specific molecular mechanisms, providing a methodological foundation for the present study.

By conducting an online GEO2R analysis of the expression profile GSE113696 in the GEO database, along with molecular biology experiments, we identified a significantly downregulated gene in CC, hsa_circ_0019686 (named circ-NOLC1). While a previous study reported its oncogenic role in epithelial ovarian cancer [19], its function in CC remains completely uncharacterized. To address this research gap, the present study aims to investigate the biological role of circ-NOLC1 in CC and explore its underlying molecular mechanisms.

## Materials and methods

### Bioinformatics analysis

Differentially expressed genes (DEGs) were identified by analyzing the GEO (http://www.ncbi.nlm.nih.gov/geo) dataset (GSE113696) using GEO2R, which utilizes the R packages GEOquery and limma for microarray data analysis. The analysis employed default parameters, including the Benjamini & Hochberg method for multiple testing correction (adjusted P-value (adj.P.Val) threshold ≤ 0.05) to screen for DEGs between HR-HPV CC tissues (Cancer group) and normal mucosal tissues (Normal group). The circBank (http://www.circbank.cn/index.html) and starBase (http://starbase.sysu.edu.cn/) databases were used to predict the miRNAs regulated by circ-NOLC1. The TargetScanHuman 7.2 (https://www.targetscan.org/vert_80/) (cumulative weighted context + + score ≤ 0.4) and miRDB (http://mirdb.org/) (score ≥ 90) databases were employed to identify targets of miR-330-5p. Additionally, the Cancer Genome Atlas (TCGA) database (https://ualcan.path.uab.edu/analysis.html) on the UALCAN website was utilized to predict the expression levels of hsa-miR-330-5p and PALM.

### Cell lines and culture conditions

Human CC [HeLa (CRM-CCL-2), CaSki (CRL-1550), C-33 A (HTB-31) and SW756 (CRL-3584)] and human normal cervical epithelial [HcerEpiC (PCS-480-011)] cell lines were obtained from the ATCC. All cell lines were cultured in the DMEM (adding 10% FBS, 1% penicillin/streptomycin (Invitrogen; Thermo Fisher Scientific, Inc) in a humidified atmosphere of 5% CO_2_ at 37˚C.

### Cell transfection

The pCD5-ciR overexpression vector for circ-NOLC1 (oe-circ-NOLC1) and the corresponding negative control (NC) plasmid (oe-NC, an empty vector lacking the target sequence) were constructed by Geneseed (Guangzhou, China). The miR-330-5p mimic oligonucleotide and its corresponding mimic-NC (mimic, 5’-UCUCUGGGCCUGUGUCUUAGGC-3’; mimic-NC, 5’-GAUGGCAUUCGAUCAGUUCUA-3’) were obtained from Ribobio (Guangzhou, China). The experimental group was transfected with the miR-330-5p mimic, while the control group was transfected with the mimic-NC. Lipofectamine 2000 (Thermo, USA) was used to transfect these overexpression plasmids (2 µg) and oligonucleotides (50nM) into HeLa or CaSki cells for 24 h at 37˚C. After transfection 24–48 h, the cells were collected for subsequent tests.

### RNA extraction and RT-qPCR

The total RNA from the CC and HcerEpiC cells was extracted using TRIzol (Invitrogen, USA). Subsequently, the cDNA was synthesized with the PrimeScript RT Reagent Kit (Takara, China). RNA expression levels were quantified using SYBR Premix Ex TaqTM (Takara). The 2^−ΔΔCq^ method was used to measure the relative expression levels of circ-NOLC1, miR-330-5p, and PALM. GAPDH served as the normalization control for circ-NOLC1 and PALM. U6 served as the normalization control for miR-330-5p. The primers for circ-NOLC1, miR-330-5p, and PALM were obtained from GenePharma (Shanghai, China), and their sequences are provided in Table 1. Primers for circ-NOLC1, PALM, U6, and GAPDH primers were designed using Primer 3 software, while the miR-330-5p primer was designed using miRNA Design software.

**Table 1:** Primers for RT-qPCR

Targets	Sequences	
hsa_circNOLC1	F: 5’-CCAATGGTAAAGCAGCCAGT-3’	R: 5’-GCGTATCGCCATCTTGGTTA-3’
U6	F: 5’-CTCGCTTCGGCAGCACA-3’	R: 5’-AACGCTTCACGAATTTGCGT-3’
miR-330-5p	F: 5’-GCGTCTCTGGGCCTGTGTC-3’	R: 5’-AGTGCAGGGTCCGAGGTATT-3’
PALM	F: 5’-CGAGGTGGACGAACTCATCC-3’	R: 5’-CTCCGCCGTGATGGTATCTTG-3’
GAPDH	F: 5’-GAAGGTGAAGGTCGGAGTC-3’	R: 5’-GAAGATGGTGATGGGATTTC-3’

### Cytoplasmic and nuclear separation

The PARIS kit (Thermo, USA) was selected to separate the total cellular fractions into cytoplasmic and nuclear components. Specific experimental methods and considerations are detailed in a previous study [20]. Briefly, cells were homogenized in cold Cell Disruption Buffer to prepare total cell lysates. The nuclear and cytoplasmic fractions were then isolated using the kit’s reagents, which allow for RNA extraction from each fraction without the need for phenol extraction or alcohol precipitation. The RNA extracted from these fractions was analyzed using RT-qPCR to measure the levels of circ-NOLC1, GAPDH (cytoplasmic control), and U6 (nuclear control).

### RNase R treatment assay

The experimental method was based on a previous report [21]. After extracting the total RNA, the RNase R treatment experiment was conducted. In brief, the total RNAs were incubated with or without RNase R (Guangzhou Geneseed Biotech. Co., Ltd.) at 37 ℃ for 30 min. The MOCK group was treated with DEPC water without RNase R. Subsequently, RT-qPCR was performed to assess the stability of circ-NOLC1.

### Cell viability assay

After transfecting the circ-NOLC1 overexpression vector or the miR-330-5p mimic, cell viability was assessed using the Super-Enhanced Cell Counting Kit-8 (Beyotime, China). Cells were prepared in DMEM medium and cultured at a density of 2 × 10^3^ cells/ml in 96-well plates. Following incubation for various time points, each well received 10 µl of CCK8 reagent and was incubated for an additional 0.5 h. Finally, cell viability was measured at an absorbance of 450 nm using the Multiskan SkyHigh (Thermo, USA).

### Scratch wound healing

Following transfection, a scratch wound healing assay was conducted to evaluate the transverse migratory capacity of CC cells. Specifically, HeLa and CaSki cells were seeded in 6-well plates (2 × 10^5^ cells per well) and were scratched using a 10 µl pipette tip to create uniform wounds. The wells were subsequently washed with phosphate-buffered saline (PBS) to remove non-adherent cells, after which medium containing 2% FBS was added. The cells were then further incubated in a 37 ˚C and 5% CO_2_ incubator. Photographs were taken at a magnification of 40 × at both 0 h and 24 h.

### Transwell assay

For the invasion assay, the transwell chambers were pre-coated with Matrigel^®^ Matrix (Corning, USA). After 24 h, the invading cells at the bottom of the chambers were fixed using 4% paraformaldehyde (Sangon, China). Subsequently, a 0.1% crystal violet solution was applied to stain the fixed CC cells. Finally, the stained CC cells were counted and photographed under a microscope for observation.

### Cell apoptosis

The flow cytometry assay was employed to observe apoptosis in HeLa and CaSki cells. Specifically, the apoptosis kit (Annexin V-FITC, BD Biosciences, USA) was used to stain the transfected CC cells. These cells were then collected and washed with PBS. Subsequently, the CC cells were suspended in 1× binding buffer and incubated with the reagents for 15 min (5 µl Annexin V-FITC/PT). Finally, the apoptosis rate was assessed using a FACScan flow cytometer analyzer (BD Biosciences).

### Xenograft mouse model

This research was approved by the Medical Ethics Committee of Harrison International Peace Hospital. All experiments involving animals were approved and conducted in accordance with the stipulations of the hospital’s Medical Ethics Committee. The 4-week-old female BALB/c nude mice (weighing 10–15 g) were procured from Charles River (Beijing, China). These mice were housed in the SPF animal laboratory, where the temperature was maintained at approximately 22 °C, humidity was kept between 50% and 60%, and a 12-hour light/dark cycle was implemented. The mice were provided with adequate food and water. Before animal modeling, nude mice were given local anesthesia. The specific steps were as follows: 1% Lidocaine HCl (local anesthetic, 2 mg/kg) was locally instilled on the back of the mouse. The degree of anesthesia in nude mice was then assessed by observing behavioral responses, gently touching the anesthetized area to evaluate pain reactions, and monitoring heart rate and respiratory rate. After the nude mouse is anesthetized, a surgical incision of 2–5 mm is made on the back of the nude mouse, and a predetermined number of tumor cells (5 × 10^6^ CaSki cells/100µl PBS per mouse) are injected into the subcutaneous tissue [22]. After surgery, wound healing of the nude mice was observed, adequate supply of food and drinking water was ensured, and tumor growth was regularly monitored every three days. The tumor size in this study did not exceed the specified maximum tumor size.

### Post-surgery care, tumor monitoring, and euthanasia procedures

After modeling, ibuprofen (30 mg/kg) was taken orally to relieve pain according to the body weight and pain level of the nude mice. Mice were randomly divided into two groups (*n* = 3 per group), including oe-NC group (CaSki cells transfected with overexpression NC plasmid) and oe-circ-NOLC1 group (CaSki cells transfected with circ-NOLC1 overexpression plasmid). In the end, the size, weight, and growth curve of the tumors were all documented. Animal care and euthanasia were carried out with the permission of the IACUC of the Harrison International Peace Hospital. The duration of the experiment was 5 weeks, and the mice were euthanized by CO_2_ inhalation. As per the 2020 Euthanasia Guidelines of the American Veterinary Medical Association (AVMA), the rate of CO_2_ replacement is 30% of the container volume per minute to ensure that the animal becomes unconscious before any pain is inflicted. After the euthanasia process, the mice tumor tissues are prepared as paraffin sections for subsequent immunohistochemistry (IHC) experiments.

### IHC

The paraffin sections of xenografts underwent torrefaction, dewaxing, and rehydration procedures. Following antigen retrieval, the sections were blocked using goat serum. Subsequently, they were incubated overnight at 4 °C with primary antibodies: anti-Ki67 (1:100, ab279653, Abcam) and anti-MMP9 (1:100, ab76003, Abcam). Next, the sections were treated with an HRP-labeled goat anti-human IgG secondary antibody (SSA001; Sino Biological Inc.) The Diaminobenzidine (DAB) developing kit (YT8204; Beijing Yita Biotech Co., Ltd.) was then used to develop the slices.

### Luciferase reporter gene detection

The pGL3-circ-NOLC1/PALM 3’-UTR-WT and pGL3-circ-NOLC1/PALM 3’-UTR-MUT luciferase reporter plasmids were obtained from GenePharma. The sequence details are presented in Table 2. HeLa and CaSki cells were transfected with these plasmids and co-transfected with either the miR-330-5p mimic or the mimic-NC using Lipofectamine 2000. Forty-eight hours post-transfection, luciferase activity was measured according to the manufacturer’s instructions for the Dual-Luciferase Reporter Assay (Promega, USA). The luciferase activity was subsequently normalized to the *Renilla* luciferase activity.

**Table 2:** Luciferase reporter plasmid binding site sequences

Gene	Type	Binding Site Sequence
circ-NOLC1	WT	CCCAGAG
circ-NOLC1	MUT	GGGUCUC
PALM 3’-UTR	WT	CCCAGAGA
PALM 3’-UTR	MUT	GGGUCUCU

Note: WT = wild-type; MUT = mutant. Sequences correspond to the binding sites for miR-330-5p in luciferase reporter plasmids

### RNA Immunoprecipitation (RIP) assay

Cell lysis was performed using the Imprint^®^ RNA Immunoprecipitation Kit (Sigma, Germany) according to the manufacturer’s instructions. Briefly, 1 mL of cell lysate (containing 1 × 10^6^ cells) was mixed with 5 µg of anti-Ago2 antibody or 5 µg of IgG isotype control, both pre-immobilized on protein-A magnetic beads. The mixture was incubated overnight at 4 °C. After incubation, the beads were washed three times with immunoprecipitation buffer. RNA associated with the beads was then extracted using TRIzol reagent, and the immunoprecipitated RNAs were quantified by RT-qPCR.

### Statistical analysis

All in vitro experiments were performed with three independent biological replicates (*n* = 3), and data were presented as mean ± standard deviation (SD); in vivo nude mouse xenograft models were conducted with *n* = 3 mice per group. Student’s *t*-test was used for two-group comparisons (normal distribution and homogeneity of variances confirmed by Shapiro-Wilk test and Levene’s test beforehand), and the Mann-Whitney U test was used when parametric assumptions were not met. For multiple-group comparisons, one-way analysis of variance (ANOVA) was performed followed by Tukey’s test for post-hoc correction, while Bonferroni correction was applied for directional hypothesis testing. Statistical significance was set at *P* < 0.05 (**P < 0.01, ***P < 0.001), and all statistical analyses were performed using GraphPad Prism 9.0.

## Results

### circ-NOLC1 is expressed at low levels in CC cells

**Fig. 1:**
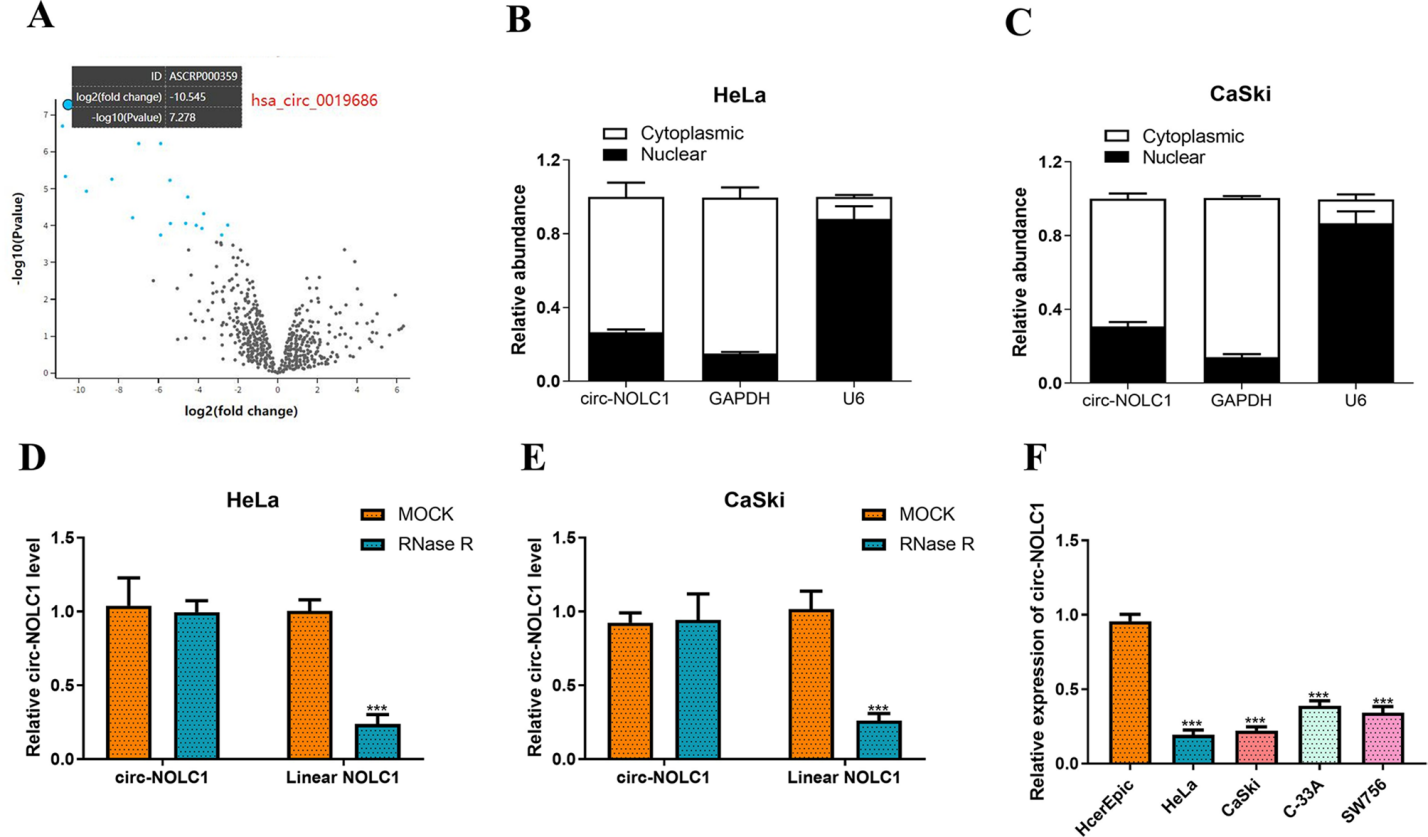
circ-NOLC1 was downregulated in CC cells. (**A**) The Gene Expression Omnibus dataset (accession no. GSE113696) analysis of differential circRNAs in CC cell lines (HeLa, CaSki, C-33 A, and SW756) compared to a normal cervical epithelial cell line (HcerEpic). Volcano map of differentially expressed genes (hsa_circ_0019686 is the most significantly downregulated gene in this dataset, adj.P.Val = 0.000246; logFC=-29.599789). RT-qPCR was used to detect the circ-NOLC1 expression level in subcellular components of (**B**) HeLa and (**C**) CaSki cells, including the nucleus (marker is U6) and cytoplasm (marker is GAPDH). The levels of circ-NOLC1 and linear NOLC1 in (**D**) HeLa and (**E**) CaSki cells treated with RNase R were detected by RT-qPCR. (**F**) RT-qPCR was applied to measure circ-NOLC1 expression in CC (HeLa, CaSki, C-33 A and SW756) and normal cervical epithelial (HcerEpic) cell lines. ***P < 0.001

We initially screened for differentially expressed circRNAs in CC by utilizing online analyses of a GEO dataset (accession no. GSE113696). As shown in Fig. 1A, we identified a circRNA, hsa_circ_001968, that was significantly downregulated in CC. Analysis of the circBank database revealed that the host gene of hsa_circ_001968 is nucleolar and coiled-body phosphoprotein 1 (NOLC1). Therefore, this study refers to this circRNA as circ-NOLC1. To determine the primary localization of circ-NOLC1 in CC cells, we conducted RT-qPCR analysis on both the nucleus and cytoplasm. The results indicated that circ-NOLC1 was predominantly located in the cytoplasm (Fig. 1B-C). To validate the loop structure of circ-NOLC1, RNase R experiments were conducted in HeLa and CaSki cells. The results demonstrated that RNase R significantly degraded linear NOLC1 (HeLa: reduced by 76%, *P* < 0.001; CaSki: reduced by 76%, *P* < 0.001), while endogenous circ-NOLC1 exhibited resistance to digestion by RNase R (Fig. 1D-E). Furthermore, the expression levels of circ-NOLC1 in normal cervical epithelial (HcerEpiC) and CC (HeLa, CaSki, C-33 A, and SW756) cell lines were examined. The data indicated that circ-NOLC1 was substantially downregulated in CC cells (HeLa: reduced by 80%, *P* < 0.001; CaSki: reduced by 77%, *P* < 0.001; C-33 A: reduced by 59%, *P* < 0.001; SW756: reduced by 64%, *P* < 0.001) when compared to the normal cells (Fig. 1F). HeLa and CaSki cells exhibited the most significant downregulation in expression. Consequently, the HeLa and CaSki cell lines were selected for the subsequent tests.

### Upregulation of circ-NOLC1 suppresses the proliferation, migration, and invasion of CC cells while facilitating their apoptosis

**Fig. 2:**
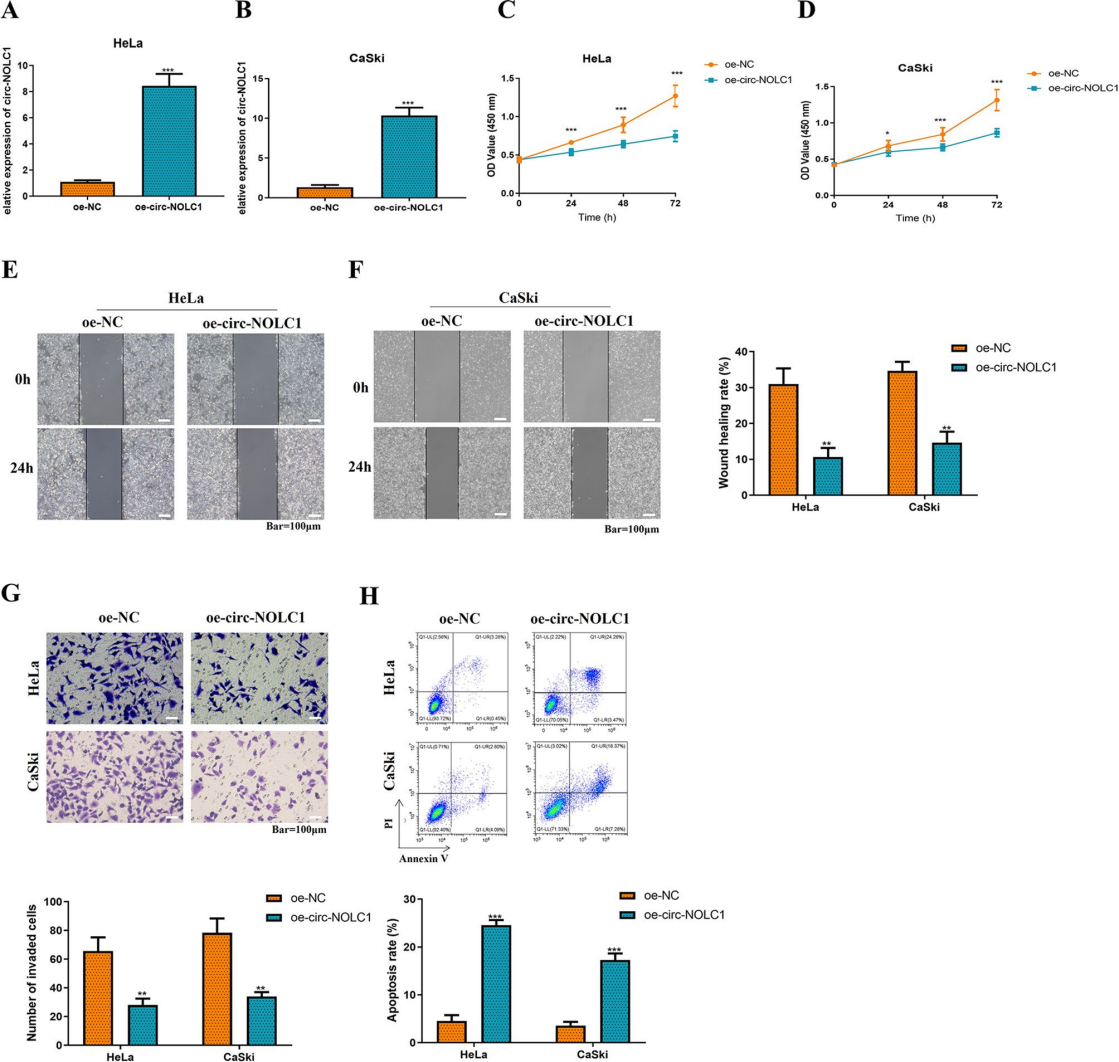
Overexpression of circ-NOLC1 inhibits the proliferation, migration, and invasion of CC cells while promoting their apoptosis. RT-qPCR was employed to assess the transfection efficiency of the circ-NOLC1 overexpression plasmid (based on the pCD5-ciR vector) in (**A**) HeLa and (**B**) CaSki cells. Cell Counting Kit-8 assay was conducted to evaluate the viability of (**C**) HeLa and (**D**) CaSki cells. A scratch wound healing assay was performed to estimate cell migration in (**E**) HeLa and (**F**) CaSki cells (scale bar, 100 μm). (**G**) Transwell assay was utilized to determine the cell invasion of HeLa and CaSki cells (scale bar, 100 μm). (**H**) Flow cytometry was used to detect the cell apoptosis rate of HeLa and CaSki cells. **P < 0.01, ***P < 0.001

To investigate the biological function of circ-NOLC1 in CC, gain-of-function experiments were conducted. First, circ-NOLC1 was overexpressed in HeLa (increased by 7.6-fold, *P* < 0.001) and CaSki (increased by 7.8-fold, *P* < 0.001) cells (Fig. 2A-B). Subsequently, the changes in cell proliferation, migration, invasion and apoptosis of HeLa and CaSki cells following circ-NOLC1 overexpression were assessed using CCK-8, wound healing, Transwell, and flow cytometry assays. As seen in Fig. 2C-D, the cell proliferation ability of HeLa (reduced by 41%, *P* < 0.001) and CaSki (reduced by 34%, *P* < 0.001) cells was dramatically decreased in the oe-circ-NOLC1 group. Correspondingly, the migration **(**Fig. 2E-F, HeLa: reduced by 66%, *P* < 0.01; CaSki: reduced by 58%, *P* < 0.01) and invasion (Fig. 2G, HeLa: reduced by 57%, *P* < 0.01; CaSki: reduced by 57%, *P* < 0.01) abilities of the oe-circ-NOLC1 group were also significantly decreased. In contrast, the apoptosis rate in the oe-circ-NOLC1 group was markedly increased (Fig. 2H, HeLa: increased by 4.4-fold, *P* < 0.001; CaSki: increased by 3.8-fold, *P* < 0.001).

### Upregulation of circ-NOLC1 suppresses the growth of CC tumors

**Fig. 3:**
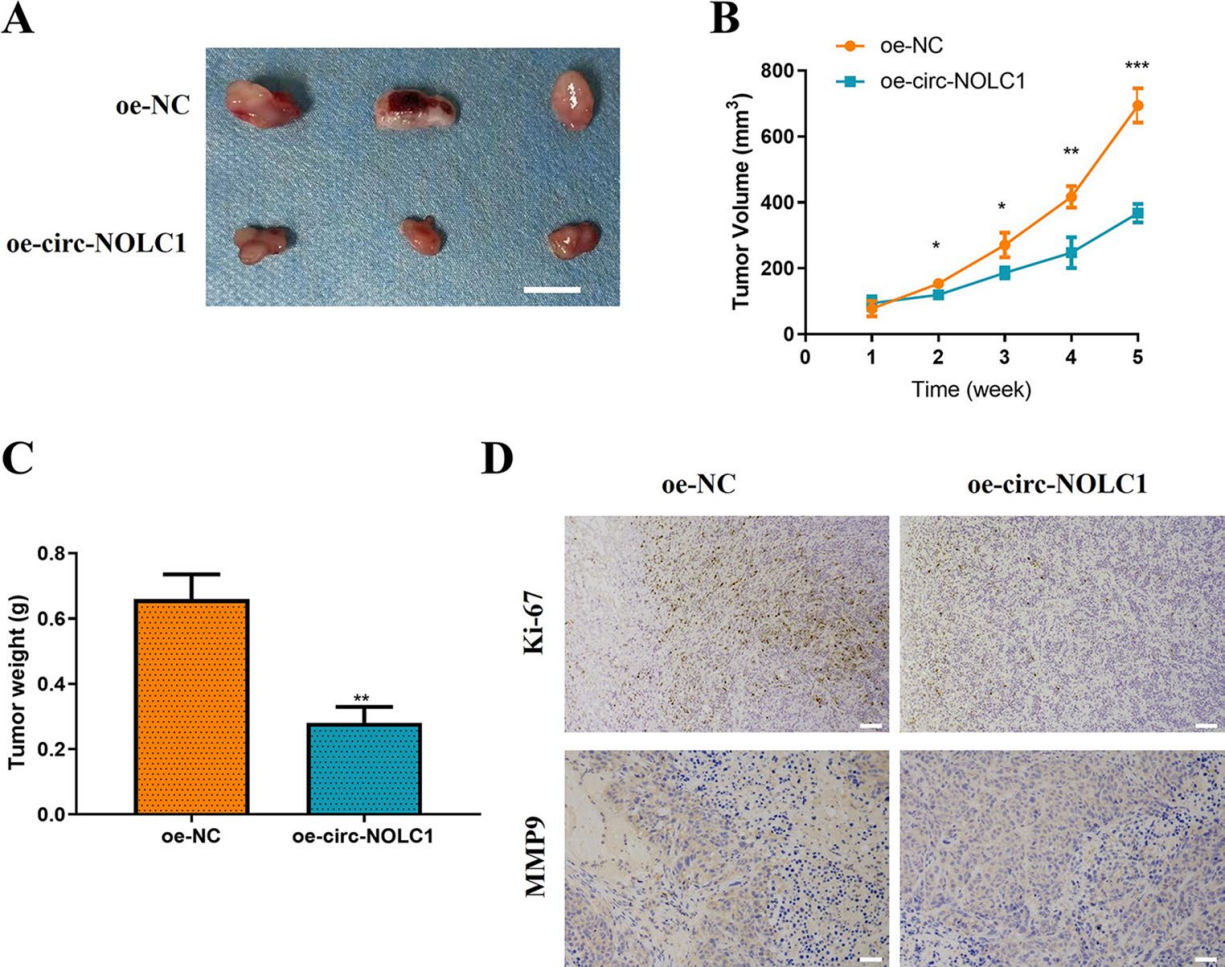
Overexpression of circ-NOLC1 inhibits the growth of CC in vivo. (**A**) Representative images from the tumorigenicity assay conducted in nude mice (*n* = 3 per group; scale bar, 1 cm). (**B**) Relative tumor growth curves. (**C**) Tumor weight plot. (**D**) The expression levels of Ki-67 and MMP9 proteins in the tumors was detected by immunohistochemistry (scale bar, 50 μm). **P < 0.01, ***P < 0.001

In vivo validation was subsequently conducted. First, a nude tumor mouse model was established using CaSki cell lines that were stably transfected with either oe-circ-NOLC1 or oe-NC. As illustrated in Fig. 3A-C, the overexpression of circ-NOLC1 in CaSki cells significantly inhibited the tumor growth of CC xenografts in nude mice (tumor volume: reduced by 47%, *P* < 0.001; tumor weight: reduced by 58%, *P* < 0.01). Next, the expression levels of Ki67 protein (a proliferation marker) and MMP9 protein (a migration marker) in the tumor tissues of the mice were analyzed using IHC. The data suggested that the upregulation of circ-NOLC1 suppressed the expression of Ki-67 and MMP9 in the tumor tissues from the CC mouse model (Fig. 3D).

### The competitive adsorption of circ-NOLC1 to miR-330-5p upregulates PALM expression

**Fig. 4:**
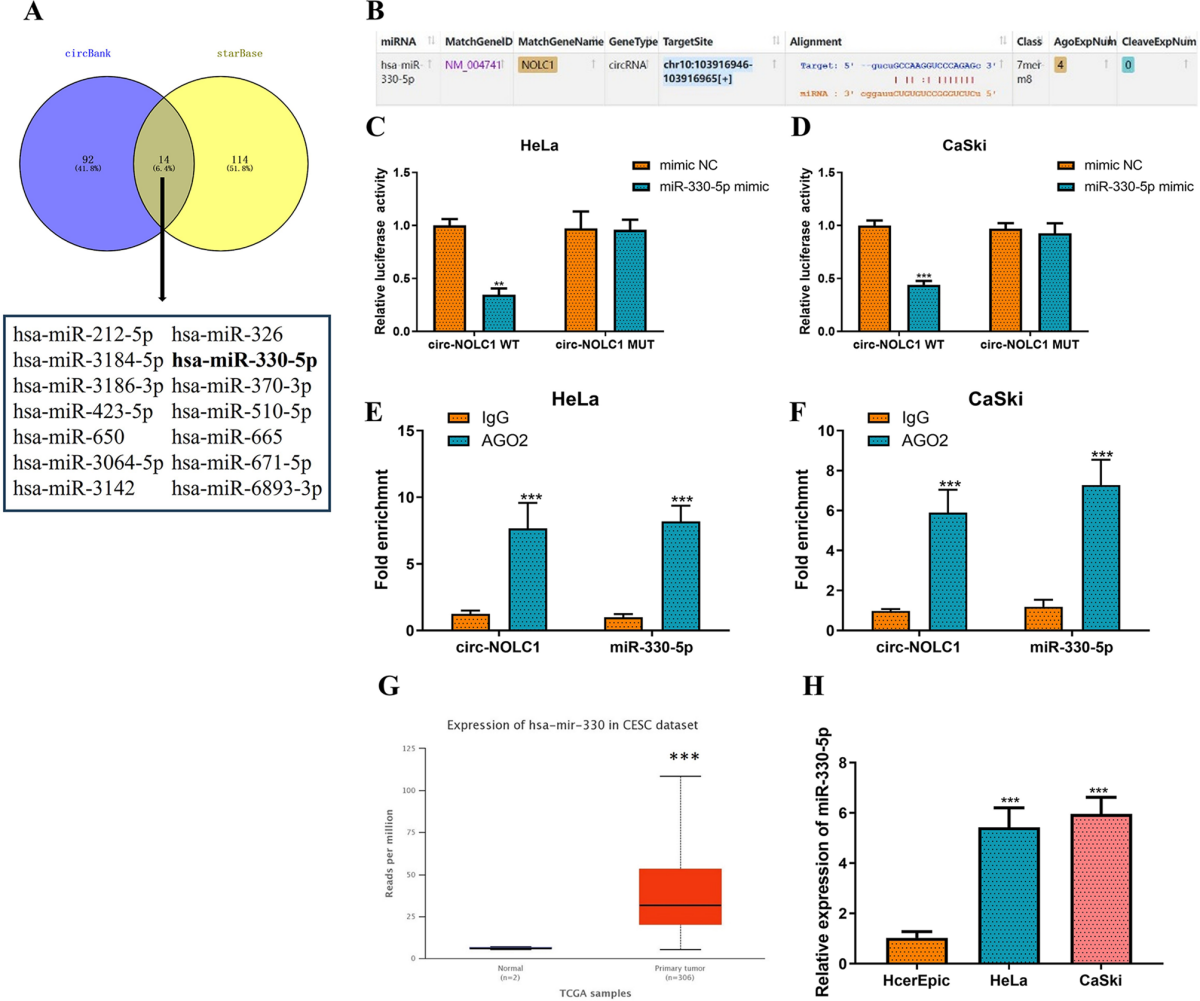
circ-NOLC1 sponges miR-330-5p. (**A**) A Venn diagram illustrating the analysis of the circBank and starBase databases to predict circ-NOLC1 target miRNAs. (**B**) Binding sites between circ-NOLC1 and miR-330-5p were predicted using the starBase database. A dual luciferase reporter gene assay was conducted to elucidate the targeting relationship between circ-NOLC1 and miR-330-5p in (**C**) HeLa and (**D**) CaSki cells. RIP assay was performed to validate the interaction between circ-NOLC1 and miR-330-5p in (**E**) HeLa and (**F**) CaSki cells. (**G**) The expression of miR-330-5p in CESC was predicted using the TCGA database (*P* = 1.62447832963153E-12). (**H**) RT-qPCR was employed to determine miR-330-5p expression in cervical cancer (HeLa and CaSki) and normal cervical epithelial (HcerEpic) cell lines. **P < 0.01, ***P < 0.001

The tests shown in Fig. 1C and D demonstrated that circ-NOLC1 predominantly exists in the cytoplasm of CC cells. This suggests that it may regulate miRNAs through an endogenous competing RNA (ceRNA) mechanism, thereby playing a regulatory role in CC. To identify the miRNAs regulated by circ-NOLC1, we first predicted them using the online databases circBank and starBase, resulting in the selection of 14 candidate miRNAs through intersection (Fig. 4A). By combining literature review with the bioinformatics analysis, hsa-miR-330-5p (miR-330-5p) was selected as the downstream miRNA of circ-NOLC1 in CC. To test our hypothesis, we predicted the sequence of circ-NOLC1 that binds to miR-330-5p using starBase (Fig. 4B). Subsequently, luciferase reporter gene assays demonstrated that circ-NOLC1 effectively sponges miR-330-5p in HeLa and CaSki cells. The data indicated that the miR‑330-5p mimic significantly suppressed the luciferase activity of the vector carrying the WT‑circ-NOLC1 site (HeLa: reduced by 65%, *P* < 0.01; CaSki: reduced by 56%, *P* < 0.001), but did not affect the luciferase activity of the vector with the mutant site (Fig. 4C-D). Using an anti-AGO2 antibody with IgG as a negative control, we conducted RIP assays in HeLa and CaSki cells. RT-qPCR analysis demonstrated that both circ-NOLC1 and miR-330-5p were significantly enriched in AGO2 immunoprecipitates compared to IgG controls (Fig. 4E-F). The analysis of the TCGA database revealed that miR-330-5p was highly expressed in CESC (Fig. 4G). Consequently, we measured the expression levels of miR-330-5p in CC cell lines using RT-qPCR. The results indicated that miR-330-5p was highly expressed in both HeLa (increased by 4.3-fold, *P* < 0.001) and CaSki (increased by 4.8-fold, *P* < 0.001) cells (Fig. 4H).

**Fig. 5:**
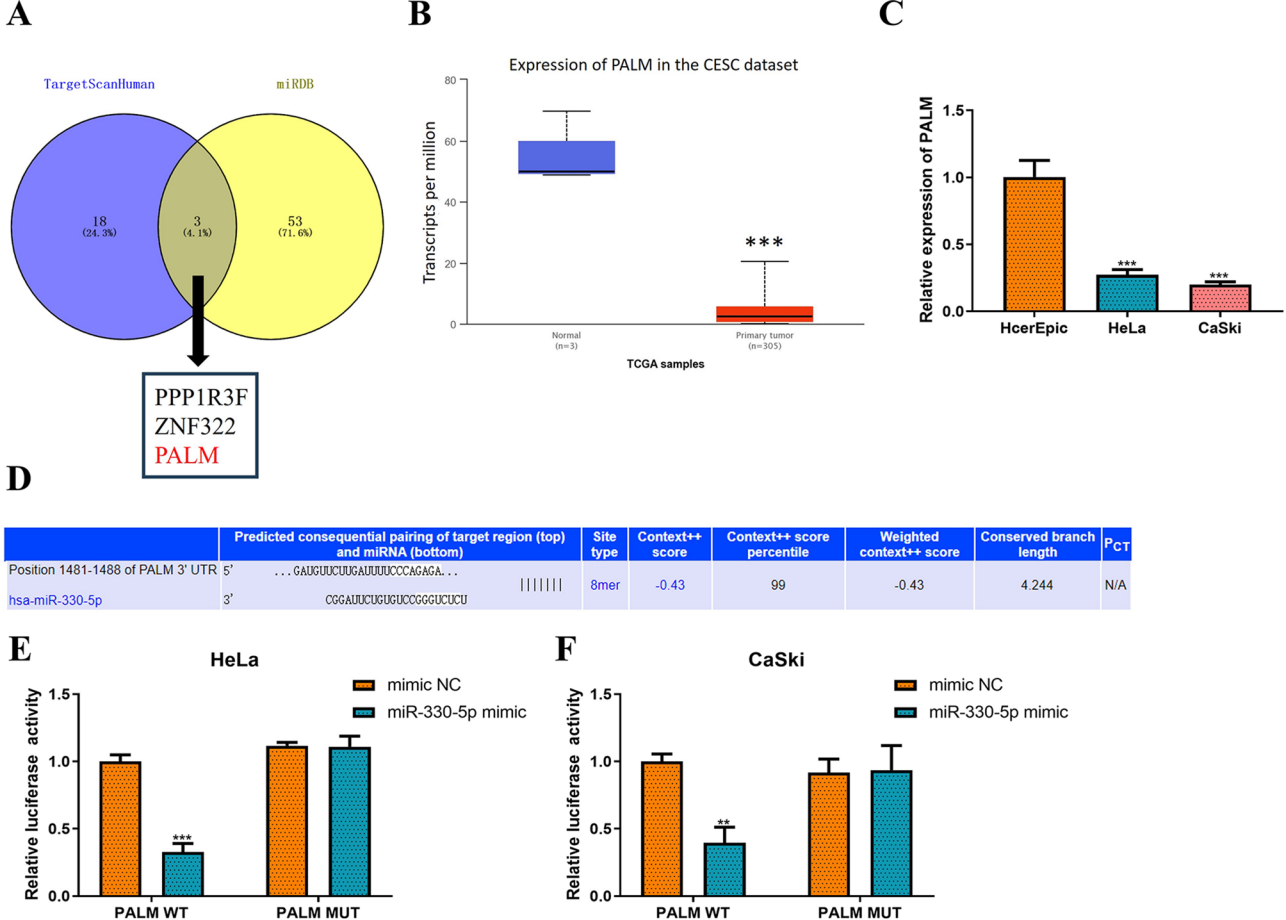
miR-330-5p interacts with PALM. (**A**) A Venn diagram illustrating the analysis of the TargetScanHuman 7.2 and miRDB databases to predict the target mRNAs of miR-330-5p. (**B**) The expression of PALM in CESC was predicted using the TCGA database (*P* = 2.14740003556813E-10). (**C**) RT-qPCR was employed to measure PALM expression in cervical cancer (HeLa and CaSki) and normal cervical epithelial (HcerEpic) cell lines. (**D**) Binding sites between miR-330-5p and PALM were predicted using the TargetScanHuman database. A dual luciferase reporter gene assay was conducted to elucidate the targeting relationship between miR-330-5p and PALM in (**E**) HeLa and (**F**) CaSki cells. **P < 0.01, ***P < 0.001

It is well known that miRNAs exert regulatory functions in various diseases by binding to the 3’-untranslated region (UTR) of mRNAs [23, 24]. Therefore, the targets of miR-330-5p were further predicted using the TargetScanHuman 7.2 (cumulative weighted context + + score ≤ 0.4) and miRDB (score ≥ 90) online databases. This analysis identified protein phosphatase 1 regulatory subunit 3 F, zinc finger protein 322 and PALM **(**Fig. 5A). By combining the literature review with the bioinformatics analysis, PALM was selected for further investigation. As seen in Fig. 5B-C, PALM was significantly downregulated in CC cells (HeLa: reduced by 73%, *P* < 0.001; CaSki: reduced by 80%, *P* < 0.001). To verify the regulation of PALM by miR-330-5p, the binding sequence of miR-330-5p to the 3’-UTR of PALM was predicted using the TargetScanHuman database (Fig. 5D). Subsequently, the interaction between miR-330-5p and PALM was elucidated through a luciferase reporter assay (Fig. 5E-F, HeLa: reduced by 67%, *P* < 0.001; CaSki: reduced by 60%, *P* < 0.01). Collectively, these results suggest that circ-NOLC1 regulates the miR-330-5p/PALM signaling axis.

### circ-NOLC1 inhibits the malignant phenotype of CC cells through the miR-330-5p/PALM signaling axis

**Fig. 6:**
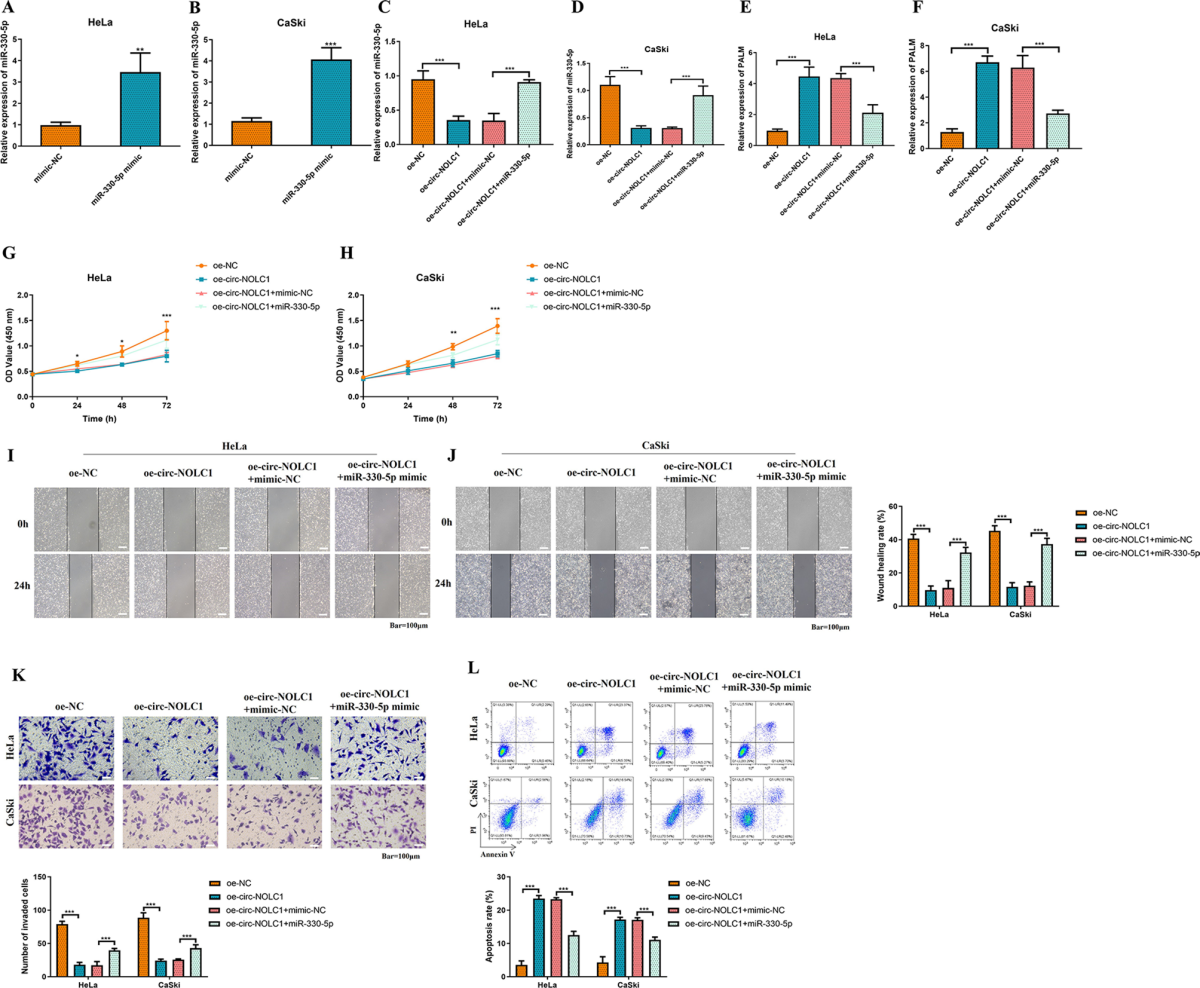
circ-NOLC1 inhibits the proliferation, migration, and invasion of CC cells while promoting apoptosis by regulating the miR-330-5p/PALM signaling axis. (**A-F**) RT-qPCR was utilized to measure miR-330-5p expression in HeLa and CaSki cells. Cell Counting Kit-8 assay was applied to evaluate the viability of (**G**) HeLa and (**H**) CaSki cells. Scratch wound healing assay was applied to estimate the migration of (**I**) HeLa and (**J**) CaSki cells (scale bar, 100 μm). (**K**) Transwell assay was used to determine the invasion of HeLa and CaSki cells (scale bar, 100 μm). (**L**) Flow cytometry was used to detect the cell apoptosis rate of HeLa and CaSki cells. **P < 0.01, ***P < 0.001

To further investigate the role of the circ-NOLC1/miR-330-5p/PALM signaling pathway in the pathological process of CC, rescue experiments were conducted. First, a miR-330-5p mimic was transfected into HeLa and CaSki cells. As seen in Fig. 6A-B, compared to the mimic-NC group, the expression levels of miR-330-5p in HeLa (increased by 2.5-fold, *P* < 0.01) and in CaSki (increased by 2.5-fold, *P* < 0.001) cells transfected with the miR-330-5p mimic were significantly upregulated. Subsequently, the circ-NOLC1 overexpression plasmid and the miR-330-5p mimic were then co-transfected to evaluate the expression of miR-330-5p (Fig. 6C-D) and PALM (Fig. 6E **and F**). As shown in Fig. 6C-D, compared with the oe-NC group, the expression level of miR-330-5p was significantly downregulated in the oe-circ-NOLC1 group (HeLa: reduced by 63%, *P* < 0.001; CaSki: reduced by 72%, *P* < 0.001). However, the expression level of miR-330-5p was significantly restored in the oe-circ-NOLC1 + mimic-NC group (HeLa: increased by 1.6-fold, *P* < 0.001; CaSki: increased by 2.0-fold, *P* < 0.001). Conversely, as shown in Fig. 6E-F, the expression level of the PALM gene was significantly upregulated in the oe-circ-NOLC1 group compared to the oe-NC group (HeLa: increased by 3.7-fold, *P* < 0.001; CaSki: increased by 4.2-fold, *P* < 0.001); in contrast, the expression level of the PALM gene was significantly downregulated in the oe-circ-NOLC1 + mimic-NC group (HeLa: reduced by 51%, *P* < 0.001; CaSki: reduced by 57%, *P* < 0.001). Additionally, the changes in proliferation, migration, invasion, and apoptosis levels of these transfected CC cells were monitored. The experimental results showed that co-transfection with oe-circ-NOLC1 and the miR-330-5p mimic counteracted the inhibitory effects of oe-circ-NOLC1 on CC cell proliferation (Fig. 6G-H, *P* < 0.001), migration (Fig. 6I-J, *P* < 0.001) and invasion (Fig. 6K, *P* < 0.001). However, co-transfection with oe-circ-NOLC1 and the miR-330-5p mimic inhibited the apoptosis-promoting effect of oe-circ-NOLC1 (Fig. 6L, *P* < 0.001).

## Discussion

CC is a type of reproductive system tumor, ranking second among gynecological malignancies in terms of incidence. In recent years, the age of onset has tended to decrease [25]. Researchers have been striving to uncover the epigenetic mechanisms underlying the pathological progression of CC [26]; however, the exact pathogenesis remains unclear. There is growing evidence that circRNAs play crucial regulatory roles in the occurrence and malignant progression of CC [8, 27]. HPV is an important factor in the induction of CC, and while it can be detected in over 99% of patients with the disease, viral infection alone is insufficient for the initiation and progression of CC [28]. Therefore, elucidating the role of small molecules in the pathogenesis and development of CC holds considerable clinical value.

The mechanisms of circRNAs in organisms are complex, with the ceRNA mechanism being the most extensively studied. Specifically, circRNAs compete for miRNA binding sites, acting as sponges for miRNAs to regulate mRNA expression [29]. It has been suggested that circRNAs could serve as novel early diagnostic markers and may represent promising therapeutic targets for tumors [14]. In CC, abnormal expression of circRNAs can disrupt the normal gene regulatory network and promote tumor growth [12, 30]. Furthermore, study has found that circRNAs can be utilized as antitumor targets in cancer therapy [31]. For instance, circRNA VPRBP inhibits CC tumorigenicity by reducing the expression of FERM domain containing 6 through sponging miR-93-5p [32]. Additionally, circ_0043280 inhibits CC growth and metastasis by regulating the miR-203a-3p/progestin and adipoQ receptor family member 3 signaling pathway [33]. circ-ITCH inhibits the proliferation, migration, and invasion of CC cells through the miR-93-5p/forkhead box K2 axis [34]. These studies suggest that different circRNAs play distinct roles in CC, exhibiting both pro-cancer and anti-cancer effects. In the present study, hsa_circ_0019686 (circ-NOLC1) was identified through an analysis of the GEO database. Bioinformatics analysis and molecular biology experiments revealed that circ-NOLC1 expression was downregulated in CC. Furthermore, the upregulation of circ-NOLC1 expression significantly inhibited the proliferation, migration, and invasion of CC cells while promoting the apoptosis rate of tumor cells. These results preliminarily indicate that circ-NOLC1 functions as a tumor suppressor gene in CC.

In the present study, we found that miR-330-5p may be regulated by circ-NOLC1. miR-330-5p is a member of the miR-330 family, which also includes miR-330-3p. These two genes exhibit both pro- and anticancer roles in various tumors [35]. For instance, miR-330-5p activates the MAPK/ERK signaling pathway by regulating the expression of SPRY2, thereby promoting the progression of liver cancer [36]. Additionally, circLRP6 enhances the expression of nuclear receptor binding protein 1 by sponging miR-330-5p, which promotes the growth and metastasis of prostate cancer [24]. Furthermore, circPTN facilitates the proliferation and stemness of gliomas by inhibiting the expression of miR-330-5p [37]. However, reports regarding the expression and function of miR-330-5p in CC have been controversial. It has been reported that the long non-coding RNA (lncRNA) EWSAT1 activates cytoplasmic polyadenylation element binding protein 4 by suppressing miR-330-5p expression, thereby accelerating the progression of CC [23]. Conversely, it has also been reported that the lncRNA WT1-AS inhibits the invasiveness of CC cells by regulating the miR-330-5p/p53 pathway [38]. In this study, integration of TCGA analysis with miR-330-5p expression profiling in CC cell lines consistently indicated that elevated levels of miR-330-5p are potentially linked to oncogenic processes in CC.

In the present study, PALM, a target gene downstream of miR-330-5p, was identified using the TargetScanHuman and miRDB databases. PALM, a phosphoprotein initially discovered in brain tissue, plays a role in regulating various biological processes, including cell movement and the maintenance of cell morphology [39]. However, the functions of PALM in tumors have been reported less frequently. A previous study showed that PALM is expressed in lymphatic endothelial cells and is closely associated with tumor lymphangiogenesis [40]. In this study, by combining TCGA analysis with the assessment of PALM expression in CC cell lines, we found that PALM acts as a tumor suppressor gene. Further rescue experiments demonstrated that the upregulation of circ-NOLC1 inhibited the proliferation, migration, and invasion of CC cells while promoting their apoptosis by activating PALM expression through a ceRNA mechanism. Based on these findings, it is plausible to hypothesize that the circ-NOLC1/miR-330-5p/PALM axis serves as a critical regulatory node in CC, potentially interacting with other signaling networks associated with cell adhesion and cytoskeletal dynamics. Furthermore, the identification of this ceRNA mechanism not only offers novel insights into the progression of CC but also underscores circ-NOLC1 as a potential prognostic biomarker and therapeutic target for restoring PALM-dependent tumor-suppressive functions in CC.

Although the specific downstream mechanisms of PALM in CC remain incompletely elucidated, its role in regulating cell motility and morphology suggests potential multifaceted effects on CC cell processes. In terms of cytoskeletal dynamics and adhesion, PALM may inhibit cell migration and invasion through pathways such as the Rho GTPase signaling or integrin-mediated adhesion; in apoptosis regulation, it might promote apoptosis via the mitochondrial apoptosis pathway or death receptor pathway; in angiogenesis and lymphangiogenesis, it could suppress relevant signaling pathways to limit tumor metastasis and growth; additionally, it may induce cell cycle arrest by regulating cyclin-related factors. Future research could delve into PALM’s downstream mechanisms through approaches such as identifying interacting proteins, analyzing phosphorylation profiles, conducting pathway inhibition assays, and validating mechanisms in vivo, thereby unlocking the therapeutic potential of the circ-NOLC1/miR-330-5p/PALM axis in CC treatment.

## Conclusion

While this study confirms that circ-NOLC1 functions as a ceRNA to sponge miR-330-5p, thereby upregulating PALM expression and inhibiting the proliferation, migration, and invasion of CC cells, as well as promoting apoptosis at both the cellular and xenograft mouse model levels, it is not without limitations. Notably, although the ceRNA mechanism is a key focus, circRNAs are known to exert regulatory functions through various other pathways, such as interactions with RBPs and the modulation of transcription, translation, or splicing events [41], which were not explored in this study. Additionally, the clinical relevance of the circ-NOLC1/miR-330-5p/PALM axis remains unvalidated, as its impact was not assessed in clinical samples or patient cohorts. Moreover, the small cohort size (*n* = 3 per group) in the xenograft mouse model may limit the statistical power and generalizability of the in vivo findings. Despite these gaps, the study establishes a novel regulatory mechanism for circ-NOLC1 in CC and highlights its potential as a diagnostic biomarker and therapeutic target for early intervention and precision treatment of CC, warranting future research to validate its clinical utility and explore broader regulatory networks.

## Data Availability

No datasets were generated or analysed during the current study.

## References

[1] Hill EK. Updates in Cervical Cancer Treatment. Clin Obstet Gynecol. 2020;63(1):3–11. 31815773 10.1097/GRF.0000000000000507

[2] Buskwofie A, David-West G, Clare CA. A review of cervical cancer: incidence and disparities. J Natl Med Assoc. 2020;112(2):229–32. 32278478 10.1016/j.jnma.2020.03.002

[3] Siegel RL, Miller KD, Jemal A. Cancer statistics, 2020. CA Cancer J Clin. 2020;70(1):7–30. 31912902 10.3322/caac.21590

[4] Fakhr E, Modic Z, Cid-Arregui A. Recent developments in immunotherapy of cancers caused by human papillomaviruses. Immunology. 2021;163(1):33–45. 33205441 10.1111/imm.13285 PMC8044335

[5] Simms KT, Hanley SJB, Smith MA, Keane A, Canfell K. Impact of HPV vaccine hesitancy on cervical cancer in japan: a modelling study. Lancet Public Health. 2020;5(4):e223–34. 32057317 10.1016/S2468-2667(20)30010-4

[6] Small W Jr., Bacon MA, Bajaj A, Chuang LT, Fisher BJ, Harkenrider MM, et al. Cervical cancer: A global health crisis. Cancer. 2017;123(13):2404–12. 28464289 10.1002/cncr.30667

[7] Cohen PA, Jhingran A, Oaknin A, Denny L. Cervical cancer. Lancet. 2019;393(10167):169–82. 30638582 10.1016/S0140-6736(18)32470-X

[8] Zhang S, Xu Y, Zheng Q. circRNA_0000285 knockdown suppresses viability and promotes apoptosis of cervical cancer cells by sponging microRNA-654-3p. Bioengineered. 2022;13(3):5251–61. 35166172 10.1080/21655979.2022.2037870 PMC8974078

[9] Chen S, Yang X, Yu C, Zhou W, Xia Q, Liu Y, et al. The potential of circrna as a novel diagnostic biomarker in cervical Cancer. J Oncol. 2021;2021:5529486. 33880120 10.1155/2021/5529486 PMC8046565

[10] Holdt LM, Kohlmaier A, Teupser D. Molecular roles and function of circular RNAs in eukaryotic cells. Cell Mol Life Sci. 2018;75(6):1071–98. 29116363 10.1007/s00018-017-2688-5 PMC5814467

[11] Zhao ZJ, Shen J. Circular RNA participates in the carcinogenesis and the malignant behavior of cancer. RNA Biol. 2017;14(5):514–21. 26649774 10.1080/15476286.2015.1122162 PMC5449088

[12] Chaichian S, Shafabakhsh R, Mirhashemi SM, Moazzami B, Asemi Z. Circular rnas: A novel biomarker for cervical cancer. J Cell Physiol. 2020;235(2):718–24. 31240697 10.1002/jcp.29009

[13] Song TF, Xu AL, Chen XH, Gao JY, Gao F, Kong XC. Circular RNA circrna_101996 promoted cervical cancer development by regulating miR-1236-3p/TRIM37 axis. Kaohsiung J Med Sci. 2021;37(7):547–61. 33728810 10.1002/kjm2.12378 PMC11896477

[14] Wu P, Li C, Ye DM, Yu K, Li Y, Tang H, et al. Circular RNA circEPSTI1 accelerates cervical cancer progression via miR-375/409-3P/515-5p-SLC7A11 axis. Aging. 2021;13(3):4663–73. 33534779 10.18632/aging.202518 PMC7906137

[15] Zhong P, Guo A, Wang L, Lin X, Feng M, Circular. RNA CDK6 suppresses cervical cancer proliferation and metastasis by sponging miR-449a. Bioengineered. 2022;13(3):4885–97. 35152839 10.1080/21655979.2022.2036898 PMC8974052

[16] Dasgupta S. Systems biology and machine learning identify genetic overlaps between lung Cancer and gastroesophageal reflux disease. OMICS. 2024;28(10):492–503. 39269895 10.1089/omi.2024.0150

[17] Cheng C, Hua J, Tan J, Qian W, Zhang L, Hou X. Identification of differentially expressed genes, associated functional terms pathways, and candidate diagnostic biomarkers in inflammatory bowel diseases by bioinformatics analysis. Experimental Therapeutic Med. 2019;18(1):278–88. 10.3892/etm.2019.7541 PMC6566124 31258663

[18] Kakar MU, Mehboob MZ, Akram M, Shah M, Shakir Y, Ijaz HW, et al. Identification of differentially expressed genes associated with the prognosis and diagnosis of hepatocellular carcinoma by integrated bioinformatics analysis. Biomed Res Int. 2022;2022:4237633. 36317111 10.1155/2022/4237633 PMC9617698

[19] Chen S, Wu W, Li QH, Xie BM, Shen F, Du YP, et al. Circ-NOLC1 promotes epithelial ovarian cancer tumorigenesis and progression by binding ESRP1 and modulating CDK1 and RhoA expression. Cell Death Discov. 2021;7(1):22. 33483472 10.1038/s41420-020-00381-0 PMC7822960

[20] Ma Y, Liu J, Yang Z, Chen P, Wang DB. CircRNA_400029 promotes the aggressive behaviors of cervical cancer by regulation of miR-1285-3p/TLN1 axis. J Cancer. 2022;13(2):541–53. 35069901 10.7150/jca.61437 PMC8771527

[21] Wang W, Luo H, Chang J, Yang X, Zhang X, Zhang Q, et al. Circular RNA circ0001955 promotes cervical cancer tumorigenesis and metastasis via the miR-188-3p/NCAPG2 axis. J Transl Med. 2023;21(1):356. 37248471 10.1186/s12967-023-04194-4 PMC10226249

[22] Ou R, Lu S, Wang L, Wang Y, Lv M, Li T, et al. Circular RNA circLMO1 suppresses cervical Cancer growth and metastasis by triggering miR-4291/ACSL4-Mediated ferroptosis. Front Oncol. 2022;12:858598. 35321435 10.3389/fonc.2022.858598 PMC8936435

[23] Zhou Q, Xie Y, Wang L, Xu T, Gao Y. LncRNA EWSAT1 upregulates CPEB4 via miR-330-5p to promote cervical cancer development. Mol Cell Biochem. 2020;471(1–2):177–88. 32556917 10.1007/s11010-020-03778-8

[24] Qin L, Sun X, Zhou F, Liu C. CircLRP6 contributes to prostate cancer growth and metastasis by binding to miR-330-5p to up-regulate NRBP1. World J Surg Oncol. 2021;19(1):184. 34158077 10.1186/s12957-021-02287-2 PMC8220703

[25] Shelar S, Shim EH, Brinkley GJ, Kundu A, Carobbio F, Poston T, et al. Biochemical and epigenetic insights into L-2-Hydroxyglutarate, a potential therapeutic target in renal Cancer. Clin Cancer Res. 2018;24(24):6433–46. 30108105 10.1158/1078-0432.CCR-18-1727 PMC6295227

[26] Laengsri V, Kerdpin U, Plabplueng C, Treeratanapiboon L, Nuchnoi P. Cervical Cancer markers: epigenetics and MicroRNAs. Lab Med. 2018;49(2):97–111. 29378033 10.1093/labmed/lmx080

[27] Song T, Xu A, Zhang Z, Gao F, Zhao L, Chen X, et al. CircRNA hsa_circrna_101996 increases cervical cancer proliferation and invasion through activating TPX2 expression by restraining miR-8075. J Cell Physiol. 2019;234(8):14296–305. 30633364 10.1002/jcp.28128 PMC13483104

[28] Kessler TA. Cervical cancer: prevention and early detection. Semin Oncol Nurs. 2017;33(2):172–83. 28343836 10.1016/j.soncn.2017.02.005

[29] Hsiao KY, Sun HS, Tsai SJ. Circular RNA - New member of noncoding RNA with novel functions. Exp Biol Med (Maywood). 2017;242(11):1136–41. 28485684 10.1177/1535370217708978 PMC5478007

[30] Yao Z, Shu L, Yi Y, Qiao L. Hsa_circRNA_000543 predicts poor prognosis and promotes cervical Cancer cell progression through regulating miR-567/ZNF268 Axis. Cancer Manag Res. 2021;13:5211–22. 34234564 10.2147/CMAR.S302201 PMC8256719

[31] Patop IL, Wust S, Kadener S. Past, present, and future of circrnas. EMBO J. 2019;38(16):e100836. 31343080 10.15252/embj.2018100836 PMC6694216

[32] Shen L, Dang J, Liu S, Xian B, Deng Y, Qu D. CircRNA VPRBP inhibits tumorigenicity of cervical cancer via miR-93-5p/FRMD6 axis. Reprod Sci. 2022;29(8):2251–64. 35501594 10.1007/s43032-022-00923-0

[33] Zhang C, Liu P, Huang J, Liao Y, Pan C, Liu J, et al. Circular RNA hsa_circ_0043280 inhibits cervical cancer tumor growth and metastasis via miR-203a-3p/PAQR3 axis. Cell Death Dis. 2021;12(10):888. 34588429 10.1038/s41419-021-04193-7 PMC8481253

[34] Li J, Guo R, Liu Q, Sun J, Wang H. Circular RNA Circ-ITCH inhibits the malignant behaviors of cervical Cancer by microRNA-93-5p/FOXK2 Axis. Reprod Sci. 2020;27(3):860–68. 31993998 10.1007/s43032-020-00140-7

[35] Jafarzadeh A, Paknahad MH, Nemati M, Jafarzadeh S, Mahjoubin-Tehran M, Rajabi A, et al. Dysregulated expression and functions of microRNA-330 in cancers: A potential therapeutic target. Biomed Pharmacother. 2022;146:112600. 34968919 10.1016/j.biopha.2021.112600

[36] Xiao S, Yang M, Yang H, Chang R, Fang F, Yang L. miR-330-5p targets SPRY2 to promote hepatocellular carcinoma progression via MAPK/ERK signaling. Oncogenesis. 2018;7(11):90. 30464168 10.1038/s41389-018-0097-8 PMC6249243

[37] Chen J, Chen T, Zhu Y, Li Y, Zhang Y, Wang Y, et al. CircPTN sponges miR-145-5p/miR-330-5p to promote proliferation and stemness in glioma. J Exp Clin Cancer Res. 2019;38(1):398. 31511040 10.1186/s13046-019-1376-8 PMC6737709

[38] Cui L, Nai M, Zhang K, Li L, Li R. LncRNA WT1-AS inhibits the aggressiveness of cervical cancer cell via regulating p53 expression via sponging miR-330-5p. Cancer Manag Res. 2019;11:651–67. 30666161 10.2147/CMAR.S176525 PMC6331070

[39] Kutzleb C, Petrasch-Parwez E, Kilimann MW. Cellular and subcellular localization of paralemmin-1, a protein involved in cell shape control, in the rat brain, adrenal gland and kidney. Histochem Cell Biol. 2007;127(1):13–30. 16847661 10.1007/s00418-006-0209-y

[40] Albrecht I, Bieri R, Leu A, Granacher P, Hagmann J, Kilimann MW, et al. Paralemmin-1 is expressed in lymphatic endothelial cells and modulates cell migration, cell maturation and tumor lymphangiogenesis. Angiogenesis. 2013;16(4):795–807. 23709172 10.1007/s10456-013-9356-7

[41] Nisar S, Bhat AA, Singh M, Karedath T, Rizwan A, Hashem S, et al. Insights into the role of circrnas: biogenesis, characterization, functional, and clinical impact in human malignancies. Front Cell Dev Biology. 2021;9:617281. 10.3389/fcell.2021.617281 PMC7894079 33614648

